# Conserved and breed-specific differences in the cervical transcriptome of sheep with divergent fertility at the follicular phase of a natural oestrus cycle

**DOI:** 10.1186/s12864-021-08060-9

**Published:** 2021-10-20

**Authors:** Laura Abril-Parreño, Kieran G. Meade, Anette Kristine Krogenæs, Xavier Druart, Sean Fair, Paul Cormican

**Affiliations:** 1 Laboratory of Animal Reproduction, Department of Biological Sciences, School of Natural Sciences, Biomaterials Research Cluster, Bernal Institute, Faculty of Science and Engineering, University of Limerick, Limerick, Ireland; 2 Animal & Bioscience Research Department, Animal & Grassland Research and Innovation Centre, Teagasc, Grange, Co. Meath, Ireland; 3 School of Agriculture and Food Science, University College Dublin, Belfield, Dublin 4, Ireland; 4 Faculty of Veterinary Medicine, Norwegian University of Life Sciences, Ås, Norway; 5 UMR 6175 INRA, CNRS-Université de Tours-Haras Nationaux, Station de Physiologie de la Reproduction et des Comportements Institut National de la Recherche Agronomique, Nouzilly, France

**Keywords:** Ovine, Cervix, RNA-sequencing, Fertility

## Abstract

**Background:**

The outcome of cervical artificial insemination (AI) with frozen-thawed semen in sheep is limited by the inability of sperm to traverse the cervix of some ewe breeds. Previous research has demonstrated that cervical sperm transport is dependent on ewe breed, as sperm can traverse the cervix in greater numbers in some higher fertility ewe breeds. However, the molecular mechanisms underlying ewe breed differences in sperm transport through the cervix remain unknown. In this study, we aimed to characterise the cervical transcriptome of four European ewe breeds with known differences in pregnancy rates following cervical AI using frozen-thawed semen at the follicular phase of a natural oestrous cycle. Cervical post mortem tissue samples were collected from two Irish ewe breeds (Belclare and Suffolk; medium and low fertility, respectively) and from two Norwegian ewe breeds (Norwegian White Sheep (NWS) and Fur; high fertility compared to both Irish breeds) at the follicular phase of a natural oestrous cycle (*n* = 8 to 10 ewes per breed).

**Results:**

High-quality RNA extracted from biopsies of the mid-region of the cervix was analysed by RNA-sequencing and Gene Ontology (GO). After stringent filtering (*P* <  0.05 and FC > 1.5), a total of 11, 1539 and 748 differentially expressed genes (DEGs) were identified in Belclare, Fur and NWS compared to the low fertility Suffolk breed, respectively. Gene ontology analysis identified significantly enriched biological processes involved in muscle contraction, extracellular matrix (ECM) development and the immune response. Gene co-expression analysis revealed similar patterns in muscle contraction and ECM development modules in both Norwegian ewe breeds, which differed to the Irish ewe breeds.

**Conclusions:**

These breed-specific biological processes may account for impaired cervical sperm transport through the cervix in sheep during the follicular phase of the reproductive cycle. This novel and comprehensive dataset provides a rich foundation for future targeted initiatives to improve cervical AI in sheep.

**Supplementary Information:**

The online version contains supplementary material available at 10.1186/s12864-021-08060-9.

## Background

The complexity of cervical anatomy in sheep usually precludes the transcervical deposition of sperm during AI. Cervical AI with frozen-thawed semen yields pregnancy rates of less than 30% worldwide [1–3]. An exception to this is in Norway, where vaginal (shot-in-the-dark) insemination with frozen-thawed semen to a natural oestrous is performed by farmers themselves and yields pregnancy rates in excess of 70% in NWS, which makes up most of their national flock [4]. The reason for the successful pregnancy rates to AI in Norway has been the focus of a number of studies by our group. Earlier studies found no differences in ram/semen parameters between countries but did identify significant ewe breed effects following cervical AI with frozen-thawed semen, with pregnancy rates of 18, 28, 44 and 77% for Suffolk, Texel, Belclare and Finnish Landrace ewes respectively [2, 5].

Further investigation into the ewe breed effects demonstrated no differences between breeds in terms of oocyte quality [6] or in hormonal profiles during the periovulatory period. Furthermore, analysis of the luteinising hormone surge and time of ovulation relative to pessary removal [7] also failed to yield discernible differences. Interestingly, while fertilisation rates following laparoscopic AI with frozen-thawed semen were similar between Suffolk and Belclare ewes, both fertilisation rates and accessory sperm number following cervical AI with frozen-thawed semen was significantly higher in Belclare than the Suffolk ewes. This demonstrates that frozen-thawed sperm can traverse the cervix in greater numbers in some ewe breeds around the time of ovulation [5]. A follow on study reported ewe breed differences in rheological characteristics of cervical mucus, which may be related to the higher number of frozen-thawed sperm penetrating cervical mucus from Belclare than that from Suffolk ewes [8]. Thus, the migration of sperm through the cervix appears to be the critical limiting factor for the success of cervical AI, especially when frozen-thawed semen is used.

A recent study by our group compared the gross anatomy (cervical length, number of cervical rings, the appearance of the external *os* type) as well as mucus production and viscosity across Irish, Norwegian and French ewe breeds at both a synchronised and a natural cycle [9]. This study did not detect a relationship between these cervical anatomical parameters or gross mucus properties and previously reported ewe breed differences in pregnancy rates following cervical AI with frozen-thawed semen. Although, there was a significant effect of phase of the cycle on mucus production, viscosity and colour. Follicular mucus was more abundant, less viscous and clearer in colour than luteal mucus, and it is in part due to the *O*-glycosylated proteins, called mucins. In a recent study, we characterized the *O*-glycan composition in the cervical mucus of six European ewe breeds at the follicular phase [10]. This was from the same animals used in the current study plus two additional breeds. We identified that the use of exogenous hormones for oestrous synchronization did not affect the *O*-glycan composition in the high fertility ewe breeds, but it did in the other ewe breeds. We also identified differences in the *O*-glycan composition between ewe breeds within the same type of oestrous cycle.

These findings point to potentially more subtle biochemical and/or molecular differences in cervical physiology. Thus, we proposed that a molecular approach is required to interrogate the cervical biology of these economically important ewe breeds to understand the mechanisms regulating sperm transport at the follicular phase of a natural oestrous cycle. A natural cycle was chosen as shot-in-the dark AI is performed to a natural oestrous in Norway, but to a synchronised oestrous in most other countries internationally. We aimed to profile the transcriptome of the cervix in two Norwegian and two Irish ewe breeds, with known differences in pregnancy rates following cervical AI, with frozen-thawed semen at the follicular phase of a natural oestrous cycle.

## Results

### Divergence in gene expression profiles most evident between Suffolk and Norwegian ewe breeds

**Fig. 1:**
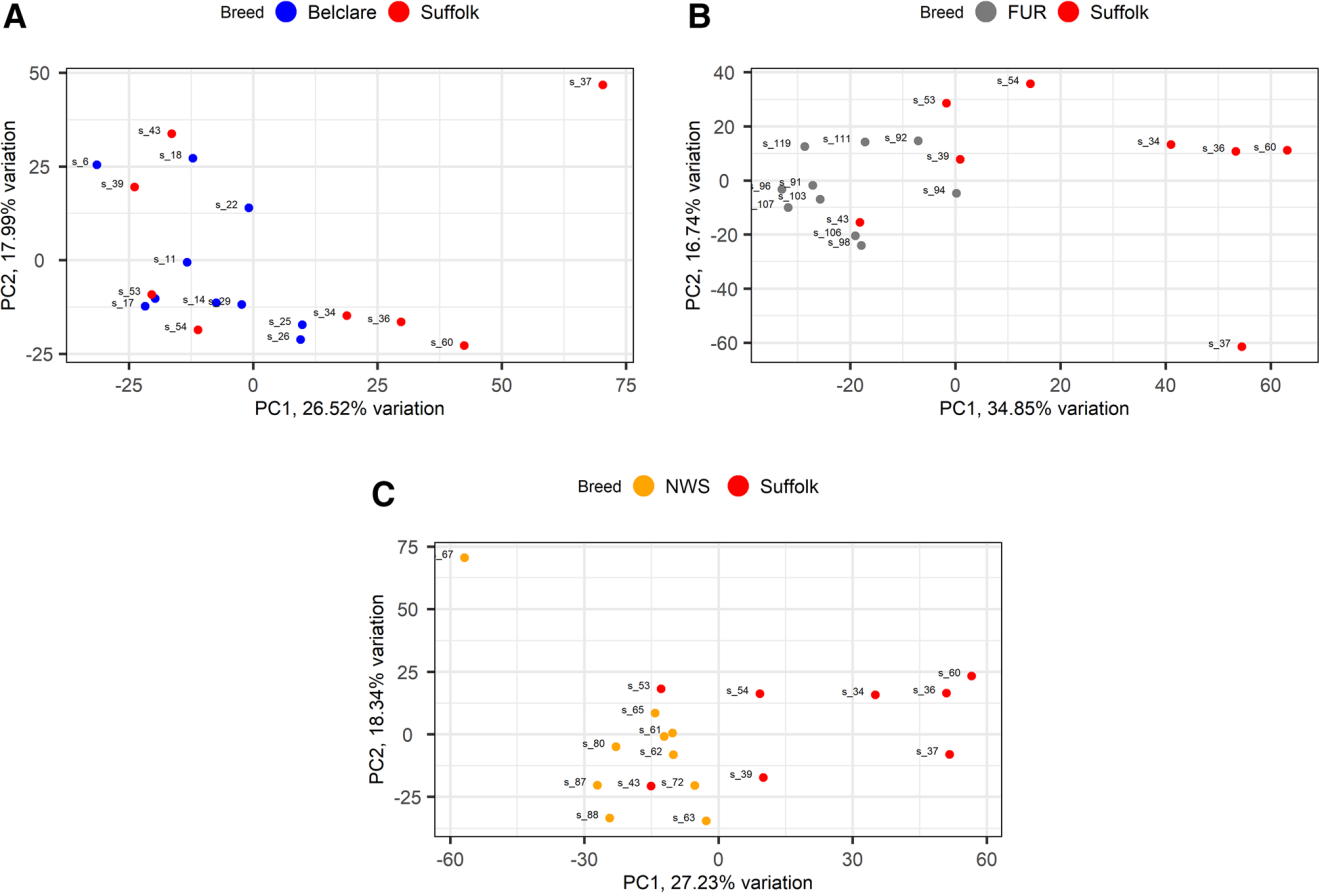
Principal component analysis (PCA) plots show distribution of RNA-sequencing samples, where colours indicate the two ewe breeds in each comparison: Belclare (**A**), Fur (**B**) and Norwegian White Sheep (NWS) (**C**) compared to Suffolk at the follicular phase of a natural oestrous cycle

RNA-sequencing data from 38 cervical biopsies of four European ewe breeds with known differences in pregnancy rates following cervical AI with frozen-thawed semen was plotted using principal component analysis (PCA) to assess the distribution of the samples within ewe breed and between ewe breeds. The Suffolk was used as a reference breed for all analyses as it has the lowest fertility. Results showed no clear segregation between the two Irish ewe breeds, whereas comparing Fur and NWS against Suffolk showed a distinct grouping as illustrated on the PCA plot (Fig. 1). This data suggested the highest fertility ewe breeds from Norway had distinct gene expression profiles compared to the low fertility Suffolk breed.

### Differential gene expression analysis identifies breed-specific differences in gene expression profiles

**Fig. 2:**
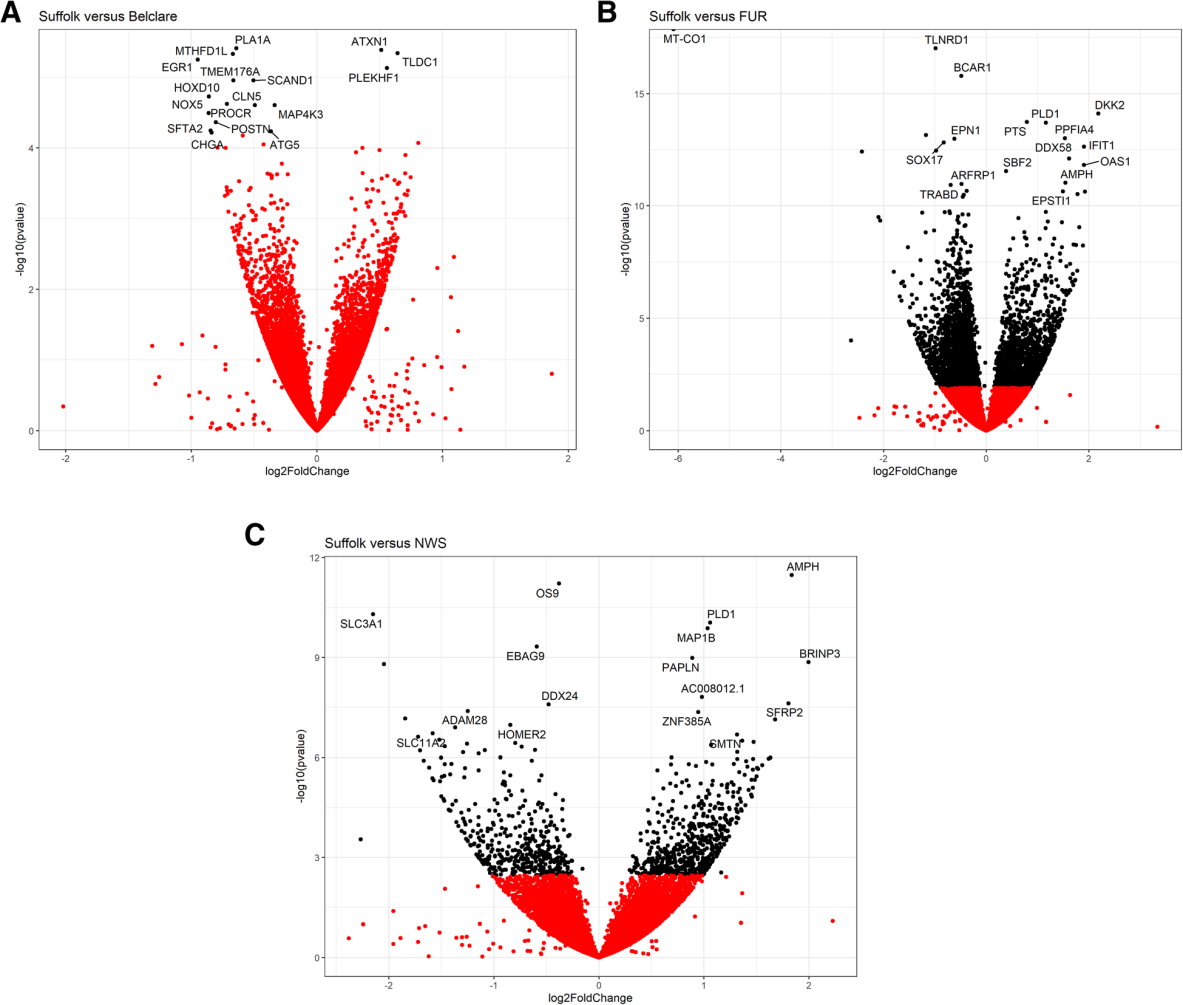
Gene expression data presented as volcano plots at the follicular phase of a natural oestrous cycle for Belclare (**A**), Fur (**B**) and Norwegian White Sheep (NWS) (**C**) compared to the low fertility Suffolk breed using log values of the FC and *P*-value. Each point represents a single gene, with those in black representing genes that survived the cut off thresholds of adjusted *P* <  0.05 and FC > 1.5 and red points represent genes with a *P* > 0.05

Using stringent statistical filtering criteria (*P* <  0.05 and FC > 1.5), data analysis identified extensive alterations in gene expression between ewe breeds at the follicular phase of a natural oestrous cycle. RNA-sequencing detected 11 DEGs (10 down-regulated and 1 gene up-regulated), 1539 (723 down-regulated and 816 up-regulated genes) and 748 (279 down-regulated and 469 up-regulated genes) in Belclare, Fur and NWS compared to the low fertility Suffolk breed (reference level), respectively. Fur had the highest difference in terms of number of DEGs compared to Suffolk breed, which is the reference level due to its lowest pregnancy rates following cervical AI using frozen-thawed semen. Volcano plots show the log2 fold-change of the top 20 DEGs in Suffolk compared to Belclare, Fur and NWS ewes (Fig. 2).

Specific gene expression differences were observed in the cervical tissue from the four ewe breeds at the follicular phase of a natural cycle. The top 5 DEGs in Belclare compared to Suffolk are shown in Supplementary Table 1. Genes such as the Early Growth Response 1 gene (*EGR1*), NADPH Oxidase 5 (*NOX5*) and Surfactant Associated 2 (*SFTA2*) were significantly up-regulated in Suffolk compared to Belclare. Only one DEG was significantly down-regulated in Suffolk compared to Belclare ewes, which was the TBC/LysM-associated domain containing 1 (*TLDC1*) gene.

A total of 1539 DEGs were up-regulated in Suffolk compared to Fur ewes, of these the top 5 are shown in Supplementary Table 2. This list included the Mitochondrially Encoded Cytochrome C Oxidase I (*COX-1*) with a FC of 68. Other genes included encoded secreted proteins such as the folate gamma receptor (*FLR3*) and the Solute Carrier Family 3 Member 1 (*SLC3A1*). The top 5 DEGs down-regulated in Suffolk compared to Fur included genes such as *DKK2* (Dickkopf WNT Signaling Pathway Inhibitor 2) and *SFRP2* (Secreted Frizzled Related Protein 2).

The top 5 DEGs of up-regulated genes in Suffolk compared to NWS included novel genes, the Folate receptor gamma (*FOLR3*) gene and the Solute Carrier Family 3 Member 1 (*SLC3A1*) gene (Supplementary Table 3). The top 5 DEGs down-regulated in Suffolk compared to NWS is also shown in Supplementary Table 3. These included genes involved in the control of the properties of the membrane associated cytoskeleton by the expression of amphiphysin (*AMPH*), the Calcium voltage-gated channel subunit alpha1 S (*CACNA1S*), Secreted Frizzled Related Protein 2 (*SFRP2*) and Protocadherin Related 15 (*PCDH15*).

### Gene ontology analysis identified enrichment of muscle development and sterol biosynthetic biological processes

No GO subcategory showed significant enrichment between Belclare and Suffolk ewes as relatively few DEGs were available for analysis. The top 5 of biological processes up-regulated and down-regulated in Fur compared to Suffolk ewes is shown in Table 1. These included down-regulated pathways involved in sterol biosynthesis and vascular transport in Fur compared to Suffolk. However, up-regulated pathways in Fur are involved in muscle contraction and development. In addition, GO analysis revealed down-regulated pathways in NWS related to anatomical structure morphogenesis, response to chemicals and stimulus (Table 1). We also identified up-regulated pathways in NWS compared to the low fertility Suffolk breed that are involved in muscle development and cell adhesion.

**Table 1:** Top 5 of biological processes enriched pathways down and up-regulated in Fur (A) and Norwegian White Sheep (NWS) (B) compared to Suffolk at the follicular phase of a natural oestrous cycle

Term name	Term ID	*P*-value
**A**		
Down-regulated in Fur compared to Suffolk		
Sterol biosynthetic process	GO:0016126	< 0.001
Cholesterol biosynthetic process	GO:0006695	< 0.001
Small molecule biosynthetic process	GO:0044283	< 0.001
Fatty acid derivative biosynthetic process	GO:1901570	< 0.001
Vascular transport	GO:0010232	< 0.001
Up-regulated in Fur compared to Suffolk		
System process	GO:0003008	< 0.001
Multicellular organismal process	GO:0032501	< 0.001
Muscle structure development	GO:0061061	< 0.001
Anatomical structure development	GO:0048856	< 0.001
Tissue development	GO:0009888	< 0.001
**B**		
Down-regulated in NWS compared to Suffolk		
Anatomical structure morphogenesis	GO:0009653	< 0.05
Response to chemical	GO:0006936	< 0.05
Response to stimulus	GO:0050896	< 0.05
Multicellular organismal process	GO:0032501	< 0.05
Positive regulation of multicellular organismal process	GO:0051240	< 0.05
Up-regulated in NWS compared to Suffolk		
System process	GO:0003008	< 0.001
Muscle structure development	GO:0061061	< 0.001
Cell adhesion	GO:0007155	< 0.001
Multicellular organismal process	GO:0032501	< 0.001
Anatomical structure development	GO:0048856	< 0.001

### Extensive differences in the immune profile of Fur and NWS compared to the low fertility Suffolk

A number of immune genes were also significantly differentially expressed between Suffolk and the Norwegian ewe breeds. Comparing the Fur to the Suffolk, the genes *IGF2* (Insulin-like growth factor 2) and *SAA1* (Serum Amyloid A1) were significantly up-regulated. In contrast, more immune genes were significantly reduced in the Suffolk compared to the Fur, and these were spread across multiple functional classes including cell-surface receptors (referred to as clusters of differentiation (CD)), Toll-like receptors (TLRs), cytokines, chemokines, acute phase proteins and genes involved in the antimicrobial response. Of particular note, *TLR1, TLR6* and multiple chemokines genes including *CXCL9, CXCL10* and *CXCL11* were all down-regulated (*P* <  0.05). Cytokines *IL17B, IL18, IL33, IL34* and *TGFβ* (Transforming Growth Factor Beta) are also reduced in expression. Lactoperoxidase (*LPO*) and multiple genes encoding members of the S100 family of calcium regulated multifunctional peptides (*S100B, S100A8, S100A9* and *S100A12*) were similarly reduced in expression. A similar profile in DEGs was evident comparing the NWS to the Suffolk. Suffolk display elevated levels of the receptors *CD14, CXCR4* and the chemokine *CXCL14* as well as increased *IGF2* and *SAA1.* Multiple CD receptors including *CD1E, CD44* and *CD274* were decreased in expression as was the expression of the chemokine *CXCL11* and multiple cytokines (*IL1A, IL17B, IL33, IL34* and *TGFβ*).

### Gene co-expression analysis across high and low fertility ewe breeds

**Fig. 3:**
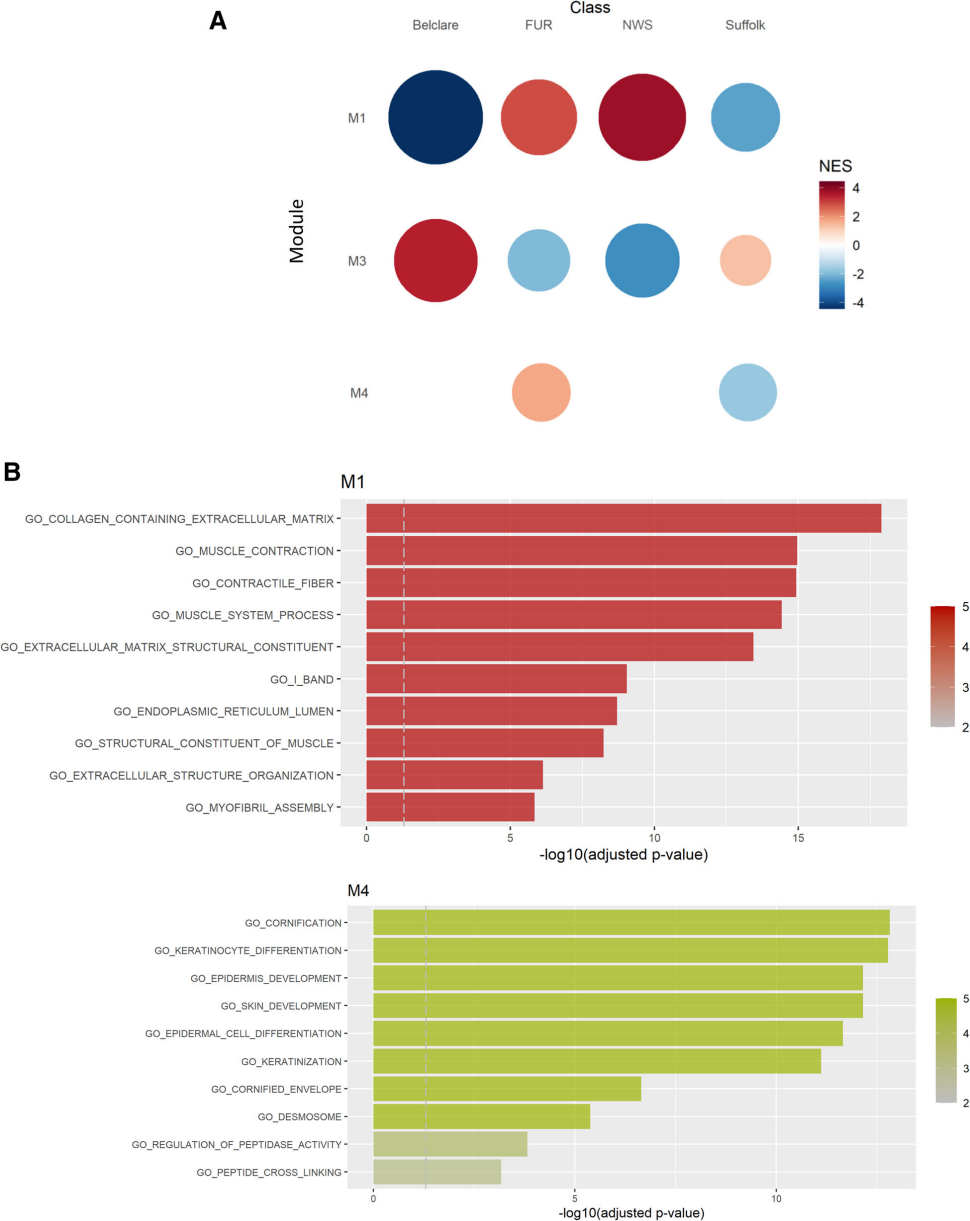
Gene co-expression analysis across the four ewe breeds (Suffolk, Belclare, Fur and NWS) at the follicular phase of a natural oestrous cycle. **A** Gene set enrichment analysis identified module 1 and 4 to differ between Irish and Norwegian ewe breeds. **B** Over-representation analysis of genes showed the gene ontology terms in module 1 and 4. The size of the circle is proportional to its normalized enriched score (NES) value. NWS = Norwegian White Sheep

RNA-sequencing data were subsequently used to perform a co-expression analysis, which allow us to identify key modules representing genes with similar expression patterns between ewe breeds. Co-expression analysis revealed six modules, from which module 1 and module 3 revealed different co-expression patterns between Irish (Suffolk and Belclare) and Norwegian ewe breeds (Fur and NWS; Fig. 3). Module 1 contained enriched pathways related to muscle contraction and ECM development, which were significantly enriched in Fur and NWS with lower levels of expression in Belclare and Suffolk ewes (*P* <  0.05; Fig. 3).

In addition, module 4 showed enriched pathways related with skin development and cell-cell junction, which had higher expression in Fur and lower expression in the low fertility Suffolk breed (*P* <  0.05). The top 5 of main regulators or hub genes of module 4 included genes involved in the keratinization process such as *KRT78* (Keratin type II cytoskeletal 78), genes encoding calcium-binding proteins that play an antimicrobial function (*S100A8, S100A9, S100A12*) and *TRIM29* (Tripartite Motif Containing 29) which is also involved in the response to viral and bacterial infections.

## Discussion

The cervix is a complex fibrous structure composed of two major types of tissue. The stromal tissue is predominantly composed of fibroblasts, smooth muscle and immune cells forming part of an ECM which is rich in collagen, hyaluronan and proteoglycans [11]. The cervical tissue also consists of epithelial cells which come in direct contact with pathogens. Therefore, an effective communication between the epithelial and immune cells in the cervical tissue is essential to maintain the integrity of the physical barrier against pathogens. Here, we hypothesised that differentially expressed genes and divergent biological processes would signpost biological phenomena that explain reasons for phenotypic differences in pregnancy rates between high and low fertility ewe breeds. We have identified for the first time extensive differences in the cervical transcriptome between high fertility Norwegian ewe breeds and the low fertility Suffolk breed. GO analysis of the DEGs in each comparison revealed novel enriched biological processes involved in muscle contraction, ECM development and immune response. In addition, co-expression analysis identified similar expression patterns related to muscle contraction and ECM development modules in both Norwegian ewe breeds, which were up-regulated compared to the low fertility Suffolk breed.

The stromal tissue (including smooth muscle and ECM) of the cervix undergoes to a cyclic remodelling across the phases of the oestrous cycle due to hormonal changes. High levels of oestradiol 2 at oestrus regulates oestrogen α (ESR1) and oxytocin receptors [12]. Oxytocin produces an increase of intracellular calcium levels in smooth muscle stimulating myometrial contractility [13]. Oxytocin also stimulates the production of PGE2 via prostaglandin endoperoxide synthase 2 (PTGS2 or COX-2), which affects both the ECM and smooth muscle of the cervical canal resulting in cervical softening and myometrial contractions [14]. Higher expression of muscle contraction and development pathways in the high fertility ewe breeds lead us to suggest that the cervix of these ewes is more susceptible to hormonal stimulation. Apart from the hormonal stimulation of the contractility, cervical smooth muscle is also responsive to inflammatory mediators [15] and even exert a reduced muscle contraction in the presence of *Chlamydia* infection as it was reported by Lee et al. (2020) [16]. Therefore, the cervical contractibility and its role in sperm transport merits further investigation.

COX-2 is involved in cervical softening and myometrial contractions [17]. However, COX-1 is involved in the maintenance of physiological events [18] in many tissues such as the gastrointestinal tract, kidneys, brain and in vascular smooth muscle of the reproductive tract [19, 20]. Our results revealed that *COX-1* was the gene with the highest difference in terms of FC between Fur and Suffolk, presenting higher levels of *COX-1* in the low fertility Suffolk breed. In the digestive system, high levels of COX-1 and low levels of COX-2 contributes to gastric mucosal defence [21, 22]. It has been described that prostaglandins produced by COX-1 regulate the mucosal blood flow and mucus secretion by the epithelial cells [22]. These studies and the higher levels of *COX-1* found in the low fertility Suffolk breed could indicate an enhanced level of mucosal defence in Suffolk which is potentially maladaptive against frozen-thawed semen.

Differences in immune status of the sheep breeds are supported by the differential expression of multiple immune relevant genes in the Norwegian breeds (high fertility) compared to the low fertility Suffolk breed. The differential expression of multiple CD markers suggests divergent cell phenotypes or activation states are present. There were higher levels of expression of *CD274* (also commonly referred to as *PDL1*) in both Norwegian ewe breeds compared to the low fertility Suffolk breed. CD274 acts to block T-cell activation and modulates the pro-inflammatory cytokine production, resulting in the suppression of cellular responses that clear the persistent human papillomavirus infection in cervical tissue [23]. The inflammatory chemokines *CXCL9, CXCL10* and *CXCL11* had higher levels in both Norwegian ewe breeds compared to Suffolk. These are predominantly involved in directing the migration of activated T-cells and natural killer cells and have been implicated in infection and inflammation [24]. It has been demonstrated that herpes simplex virus induces *CXCL9* expression in human cervical epithelial cells [25] as well as *CXCL10* in mice cervical cells [26], although its role in healthy cervical cells has not been studied yet. We identified the expression of genes encoding antimicrobial peptides such as the S100 proteins, a family of calcium-binding cytosolic proteins that have a broad range of intracellular and extracellular functions through regulating calcium balance, cell recruitment and inflammation [27, 28]. Members of the S100 family such as *S100A8, S100A9, S100B* and *S100A12* were up-regulated in the high fertility ewe breeds, suggesting higher protection against bacteria in the cervix of these ewe breeds. Proteomics studies using cervicovaginal mucus from women also detected the presence of members of the S100 family [29, 30]. In addition, Foley et al. (2015) [31] observed increased levels of *S100A9* in endometrial biopsies of healthy cows compared to the group of cows with clinical endometriosis. Differential expression of specific TLRs, including *TLR1* and *TLR6* which detect lipopeptides, may hint at differences in the cervical microbiome between breeds [32]. Collectively, these results support a divergent immune response between ewe breeds, and implicate the regulation of inflammation as a key process differentiating the cervical transcriptomic response. Sub-optimal protection against bacteria in the cervix of the Suffolk may be one process which contributes to poorer reproductive outcomes.

Results from the co-expression analysis revealed that pathways involved in cell-cell junction and desmosome had higher expression in Fur and lower expression in the low fertility Suffolk breed. The structure and configuration of the tight junctions has been reported to be modified by cytokines such as TNF*α* and TGF*β* in epithelial cell lines [33], including uterine epithelium [34]. Although its role in the tight junctions between cervical epithelial cells has not been reported, we identified higher expression of *TGFβ* in high fertility ewe breeds compared to Suffolk, which could be indicative of good integrity of the cervical barrier in these ewe breeds thereby protecting sperm as they progress along the cervix.

## Conclusions

In conclusion, the present study has defined, for the first time, the cervical transcriptome profile of four European ewe breeds with known differences in pregnancy rates following cervical AI with frozen-thawed semen. We identified extensive alterations in the cervical expression between high and low fertility ewe breeds, which were mainly involved in muscle contraction, ECM development and immune response. Further investigation in some specific genes related to the enriched pathways presented above as well as more functional studies of the products of these gene could assist in the improvement of sperm transport through the cervix following cervical AI with frozen-thawed semen and ultimate improve sheep fertility.

## Methods

### Ethical approval

Protocols were developed in accordance with the Cruelty to Animals Act (Ireland 1876, as amended by European Communities regulations 2002 and 2005) and the European Community Directive 86/609/EC. In Norway the study was approved by Norwegian Food safety Authority (FOTS ID 13168). In Ireland, all animal procedures were conducted under experimental license from the Health Products Regulatory Authority and the study was approved by the Teagasc animal ethics committee. This study was carried out in compliance with the ARRIVE Guidelines for reporting animal research [35].

### Experimental design and tissue collection

The animal model has previously described by Abril-Parreño et al. (2021) [9] since this experiment was performed as a part of larger study, which aimed to interrogate the ewe breed effect on mucus properties and anatomical characteristics across the oestrous cycle at both a synchronised and a natural oestrous. All the ewes used in this study were multiparous in a range of 4 to 5 years old. In this experiment, we examined the gene expression of the sheep cervix of four ewe breeds across two countries: Ireland (Belclare and Suffolk; high and low fertility, respectively) and Norway (NWS and Fur; both with high fertility compared to the Irish ewe breeds) at the follicular phase of a natural cycle. We used these ewe breeds due to their known different pregnancy rates following cervical/vaginal AI with frozen-thawed semen. Suffolk ewes were the reference level (negative control) in this study as they have the lowest reported pregnancy rates [2]. Cervical tissue samples were collected post-mortem from the four ewe breeds at the follicular phase of a natural cycle (*n* = 8 to 10 ewes per breed) approximately 12 h after fist observed in standing oestrus. All ewes were checked twice daily for signs of oestrus over a 6 day period using a teaser ram with an apron fitted (no semen/seminal plasma was allowed to be deposited into the vagina of the ewe). Following euthanasia, the ovaries were assessed for the presence of an active corpus luteum (luteal phase) or dominant follicles (follicular phase). The reproductive tracts were then longitudinally opened and two sections were taken from the mid region of the cervix. The mid-region of the cervix was defined as the region between the first and the last cervical ring. The cervix is a rugged structure and therefore the folds were avoided.. All samples were snap-frozen in liquid nitrogen, and subsequently stored at − 80 °C until RNA isolation.

### Tissue processing and RNA extraction

Frozen cervical tissue immersed in TRIzol reagent was homogenized using a homogenizer (Bio-gen Pro200 Homogenizer, Pro Scientific) in order to lyse the tissue. The RNA extraction was completed using the RNeasy Kit (Quiagen Ltd., Crawley, West Sussex, UK) according to the manufacturer’s instructions. Total RNA concentration was quantified using the Nanodrop ND-1000 UV-Vis Spectophotometer (NanoDrop Technologies Inc., Wilmington, DE, USA). Quality of RNA was ascertained with the use of 2100 Agilent Bioanalyzer (Agilent Technologies, Santa Clara, CA, USA). RNA integrity number (RIN) was greater than 7 in all samples and RNA aliquots were stored at − 80 °C after extraction.

### Library preparation and RNA-sequencing

RNA libraries were prepared for a total of 38 cervical tissue samples (Illumina® TruSeq® Stranded mRNA Library preparation Kit) to convert mRNA into cDNA libraries for DNA sequencing. Briefly, this involved isolation of poly-A tailed mRNA using poly-T oligo attached magnetic beads, reverse transcription to form double stranded cDNA and the ligation of indexing adaptors. Indexes were allocated to specific samples prior to library construction so that each sample within a pool had a unique bar code. Following adapter ligation, DNA fragments were selectively enriched by performing PCR. Quality control checks were performed to assess the quality and quantity of the ds cDNA libraries. The Agilent 2100 Bioanalyzer (Agilent Technologies) was used to assess purity of the samples, using the Agilent DNA 1000 kit. Library quantity was measured using the Qubit fluorometer. These steps were previously reported by Brewer et al. 2020 [36]. All libraries were sequenced on an Illumina NovaSeq sequencer by Macrogen, Inc. (Seoul, Republic of Korea) where they were sequenced using an Illumina NovaSeq. Sequencing was performed for each sample at 2 × 150 bp paired end reads (50 M reads).

### Differential expression analysis

Quality assessment of the raw sequence data was carried out using the software FastQC (v 0.11.8; http://www.bioinformatics.babraham.ac.uk/projects/fastqc/). Data were quality and adapter trimmed using the BBDuk java package to trim Illumina adapter sequences and any low quality bases (Phred score < 20) from the 3′ end of sequence read pairs. Reads were aligned to the ovine genome Oar_v3.1 using the Spliced Transcripts Alignment to a Reference (STAR) aligner. A maximum of two mismatches with the reference genome were allowed and only uniquely mapped read pairs were retained for downstream analysis. Read counts overlapping all protein coding genes in the Oar_v3.1 Ensembl (v.95) annotation were estimated using featureCounts. To filter out lowly expressed genes, genes with less than one count per million in at least ten samples were discarded from the analysis. Remaining gene counts were normalized uses the median of ratios method as implemented in DeSeq2 (version 1.130.0) [37] to account for varying sequencing depth between samples. Transcript counts were modelled by fitting the data to a negative binomial distribution using genewise dispersion estimates and differentially expressed genes were identified with a generalized linear model likelihood ratio test. Statistical tests were corrected for multiple testing using the Benjamini-Hochberg method. DEGs with an adjusted *P* <  0.05 and a log2 FC threshold of 1.5 were used for further differentially expressed gene data exploration and pathway analysis.

### Functional and pathway enrichment analysis

The gProfiler2 (v. 0.2.0) package was used to identify aggregated functional profiles of genes and gene clusters in the DEG lists. GO terms and Reactome pathways were analysed with an enrichment threshold cut-off of *P* <  0.05. The R package rrvgo (v.1.1.4) was used to reduce the redundancy of significantly enriched GO terms by grouping similar terms based on their similarity within the GO hierarchy. Gene co-expression network analyses was carried out using the R package Cemitools (v1.14.0). For any modules identified a gene set enrichment analysis was carried out to indicate if each module was induced or repressed in the different ewe breeds. Finally, an over representation analysis was implemented to identify enriched biological functions in each module.

## Supplementary Information

**Additional file 1: Table S1.** Top 5 differentially expressed genes (up and down-regulated) in Suffolk compared to Belclare. The genes shown in these tables were found to be significant with a *P* <  0.05 and FC > 1.5. **Table S2.** Top 5 differentially expressed genes (up and down-regulated) in Suffolk compared to Fur. The genes shown in these tables were found to be significant with a *P* <  0.05 and FC > 1.5.**Table S3.** Top 5 differentially expressed genes (up and down-regulated) in Suffolk compared to Norwegian White Sheep (NWS). The genes shown in these tables were found to be significant with a *P* <  0.05 and FC > 1.5.

**Additional file 2: Table S4.** Mapping information. **Table S5.** Differentially expressed genes (DEGs) in Suffolk compared to Belclare. **Table S6.** Differentially expressed genes (DEGs) in Suffolk compared to Fur. **Table S7.** Differentially expressed genes (DEGs) in Suffolk compared to Norwegian White Sheep (NWS).

**Additional file 3: Table S8.** List of biological processes up-regulated in Fur compared to Suffolk. **Table S9.** List of biological processes down-regulated in Fur compared to Suffolk. **Table S10.** List of biological processes up-regulated in Norwegian White Sheep (NWS) compared to Suffolk. **Table S11.** List of biological processes down-regulated Norwegian White Sheep (NWS) compared to Suffolk.

## Abbreviations

AI: Artificial Insemination
DEG: Differentially Expressed Gene
ECM: Extracellular Matrix
FC: Fold Change
GO: Gene Ontology
NWS: Norwegian White Sheep
PCA: Principal Component Analysis
PCR: Polymerase Chain Reaction
RIN: RNA Integrity Number
STAR: Spliced Transcripts Alignment to a Reference
