# Supplementary material for: Conserved and breed-specific differences in the cervical transcriptome of sheep with divergent fertility at the follicular phase of a natural oestrus cycle
Source: BMC Genomics. 2021 Oct 20;22:752. doi: 10.1186/s12864-021-08060-9 (PMC8527727; doi:10.1186/s12864-021-08060-9)
Supplement: Supplementary file 1 — Additional file 1: Table S1. Top 5 differentially expressed genes (up and down-regulated) in Suffolk compared to Belclare. The genes shown in these tables were found to be significant with a P < 0.05 and FC > 1.5. Table S2. Top 5 differentially expressed genes (up and down-regulated) in Suffolk compared to Fur. The genes shown in these tables were found to be significant with a P < 0.05 and FC > 1.5.Table S3. Top 5 differentially expressed genes (up and down-regulated) in Suffolk compared to Norwegian White Sheep (NWS). The genes shown in these tables were found to be significant with a P < 0.05 and FC > 1.5. [file 12864_2021_8060_MOESM1_ESM.docx]

**Additional File 1:** **Conserved and breed-specific differences in the cervical transcriptome of sheep with divergent fertility at the follicular phase of a natural oestrus cycle**

Authors: Laura Abril-Parreño^1, 2^, Kieran G. Meade^3^, Anette Kristine Krogenæs^4^, Xavier Druart^5^, Sean Fair^1,* ¶^ and Paul Cormican^2, ¶^

**Table S1**. Top 5 differentially expressed genes (up and down-regulated) in Suffolk compared to Belclare. The genes shown in these tables were found to be significant with a *P < 0.05* and FC > 1.5.

| Gene symbol | Ensembl ID | Gene name | Gene function |
| --- | --- | --- | --- |
| Up-regulated in Suffolk compared to Belclare | | | |
| *EGR1* | ENSOARG00000016470 | Early Growth Response 1 | - C_2_H_2_-type zinc-finger proteins  - Transcriptional regulator  - Differentiation and mitogenesis |
| *NOX5* | ENSOARG00000018585 | NADPH Oxidase 5 | - Calcium-dependent NADPH oxidase that generates superoxide  - Calcium-dependent proton channel that may regulate redox-dependent processes in lymphocytes and sperm |
| *HOXD10* | ENSOARG00000017514 | Homeobox D10 | - DNA-binding transcription factor activity  - Chromatin binding |
| *SFTA2* | ENSOARG00000010735 | Surfactant Associated 2 | - Putative surfactant protein |
| *CHGA* | ENSOARG00000013347 | Chromogranin A | - Produces the precursor to peptides such as catestatin and chromofungin (antimicrobial activity and antifungal activity, respectively) |
| Down-regulated in Suffolk compared to Belclare | | | |
| *TLDC1* | ENSOARG00000010901 | TBC/LysM-associated domain containing 1 | -Protein binding  -Regulation of cell migration and proliferation |

**Table S2**. Top 5 differentially expressed genes (up and down-regulated) in Suffolk compared to Fur. The genes shown in these tables were found to be significant with a *P < 0.05* and FC > 1.5.

| Gene symbol | Ensembl ID | Gene name | Gene function |  |
| --- | --- | --- | --- | --- |
| Up-regulated in Suffolk compared to Fur | | | |  |
| *COX-1* | ENSOARG00000000016 | Mitochondrially Encoded Cytochrome C Oxidase I | - Component of the cytochrome C oxidase, the last enzyme in the mitochondrial electron transport chain which drives oxidative phosphorylation  - Enzyme in the synthesis of prostaglandins and *COX* inhibitors have been shown to have an effect on female fertility | |
| *FOLR3* | ENSOARG00000004127 | Folate receptor gamma | - Folic acid transport  - Neutrophil degranulation | |
| Novel gene | ENSOARG00000002631 | - | - | |
| Novel gene | ENSOARG00000016098 | - | - | |
| *SLC3A1* | ENSOARG00000006308 | Solute Carrier Family 3 Member 1 | -Type II membrane glycoprotein  - Amino acid transport  - L-cysteine transport  - Carbohydrate metabolic process | |
| Down-regulated in Suffolk compared to Fur | | | | |
| *DKK2* | ENSOARG00000008961 | Dickkopf WNT Signaling Pathway Inhibitor 2 | - Multicellular organism development.  -Regulation of canonical WNT signalling pathway | |
| *SFRP2* | ENSOARG00000001680 | Secreted Frizzled Related Protein 2 | - Modulator of WNT signalling  - Endopeptidase activator activity  - Fibronetin and integrin binding | |
| *OAS1* | ENSOARG00000002881 | 2'-5'-Oligoadenylate Synthetase 1 | -Role in cellular innate antiviral response  -Glucose homeostasis and metabolic process  -Interferon-gamma-mediated signalling pathway | |
| *IFIT1* | ENSOARG00000015177 | Interferon Induced Protein With Tetratricopeptide Repeats 1 | -Response to virus  -Type I interferon signalling pathway | |
| *CHRM2* | ENSOARG00000001210 | Cholinergic Receptor Muscarinic 2 | - Phospholipase C activity, leading to the release of inositol trisphosphate; this then triggers calcium ion release into the cytosol.  - Response to virus | |

**Table S3**. Top 5 differentially expressed genes (up and down-regulated) in Suffolk compared to Norwegian White Sheep (NWS). The genes shown in these tables were found to be significant with a *P < 0.05* and FC > 1.5.

| Gene symbol | Ensembl ID | Gene name | Gene function |
| --- | --- | --- | --- |
| Up-regulated in Suffolk compared to NWS | | | |
| *FOLR3* | ENSOARG00000004127 | Folate receptor gamma | - Folic acid transport  - Neutrophil degranulation |
| *SLC3A1* | ENSOARG00000006308 | Solute Carrier Family 3 Member 1 | -Type II membrane glycoprotein  - Amino acid transport  - L-cysteine transport  - Carbohydrate metabolic process |
|  | ENSOARG00000006419 | Major allergen Equ c 1-like | -Molecule binding |
|  | ENSOARG00000004815 | Mast cell protease 3-like | -Proteolysis |
| Novel gene | ENSOARG00000016098 | - | - |
| Down-regulated in Suffolk compared to NWS | | | |
| *BRINP3* | ENSOARG00000010120 | BMP/Retinoic Acid Inducible Neural Specific 3 | -Cell cycle arrest  -Cellular response to retinoic acid  -Negative regulation of mitotic cell cycle |
| *AMPH* | ENSOARG00000017838 | Amphiphysin | -Control of the properties of the membrane associated cytoskeleton |
| *CACNA1S* | ENSOARG00000017449 | Calcium voltage-gated channel subunit alpha1 S | -Pore-forming, α-1S subunit of the voltage-gated calcium channel  -Triggers Ca^2+^ release and muscle contraction |
| *SFRP2* | ENSOARG00000001680 | Secreted Frizzled Related Protein 2 | - Modulator of WNT signalling.  - Endopeptidase activator activity  -Fibronectin and integrin binding |
| *PCDH15* | ENSOARG00000013216 | Protocadherin Related 15 | - Calcium-dependent cell-cell adhesion  - Expressed in cytotoxic tumor-derived T- and NK-cell lines |
